# cFOS as a biomarker of activity maturation in the hippocampal formation

**DOI:** 10.3389/fnins.2023.929461

**Published:** 2023-07-13

**Authors:** Maria Pompeiano, Matthew T. Colonnese

**Affiliations:** ^1^Department of Pharmacology and Physiology, The George Washington University, Washington, DC, United States; ^2^Departamento de Bioingeniería, Universidad Carlos III de Madrid, Madrid, Spain

**Keywords:** cFOS, waking, hippocampus, entorhinal cortex, septum, anterior thalamic nuclei, head direction system, theta rhythm

## Abstract

We explored the potential for cFOS expression as a marker of functional development of “resting-state” waking activity in the extended network of the hippocampus and entorhinal cortex. We examined sleeping and awake mice at (P)ostnatal days 5, 9, 13, and 17 as well as in adulthood. We find that cFOS expression is state-dependent even at 5 days old, with reliable staining occurring only in the awake mice. Even during waking, cFOS expression was rare and weak at P5. The septal nuclei, entorhinal cortex layer (L)2, and anterodorsal thalamus were exceptional in that they had robust cFOS expression at P5 that was similar to or greater than in adulthood. Significant P5 expression was also observed in the dentate gyrus, entorhinal cortex L6, postsubiculum L4-6, ventral subiculum, supramammillary nucleus, and posterior hypothalamic nucleus. The expression in these regions grew stronger with age, and the expression in new regions was added progressively at P9 and P13 by which point the overall expression pattern in many regions was qualitatively similar to the adult. Six regions—CA1, dorsal subiculum, postsubiculum L2-3, reuniens nucleus, and perirhinal and postrhinal cortices—were very late developing, mostly achieving adult levels only after P17. Our findings support a number of developmental principles. First, early spontaneous activity patterns induced by muscle twitches during sleep do not induce robust cFOS expression in the extended hippocampal network. Second, the development of cFOS expression follows the progressive activation along the trisynaptic circuit, rather than birth date or cellular maturation. Third, we reveal components of the egocentric head-direction and theta-rhythm circuits as the earliest cFOS active circuits in the forebrain. Our results suggest that cFOS staining may provide a reliable and sensitive biomarker for hippocampal formation activity development, particularly in regard to the attainment of a normal waking state and synchronizing rhythms such as theta and gamma.

## Introduction

The hippocampus and entorhinal cortex are critical components of the brain's memory and navigation systems. As a major hub in the adult and developing brain, it is no surprise that hippocampus dysfunction is implicated in major neurodevelopmental disorders (Griguoli and Cherubini, 2017). The developing hippocampal formation displays age-specific patterns of electrical activity that are necessary for the maturation of neural circuits (Cossart and Khazipov, 2022). In acute slice preparations, “synchronous plateau assemblies” (SPAs) are the earliest form of coordinated activity. These appear in the hippocampus soon after birth and are driven by gap junction-coupled neurons. SPAs recruit increasing numbers of neurons before being replaced by “giant depolarizing potentials” (GDPs) by the end of the first post-natal week. GDPs are synaptic events driven by the excitatory action of GABA (Ben Ari, 2001). *In vivo*, hippocampal activity in the week after birth consists largely of immature/early sharp waves largely triggered by myoclonic twitches during sleep, which enter the hippocampus through the entorhinal cortex (Del Rio-Bermudez and Blumberg, 2022; Leprince et al., 2023). Hippocampal activity undergoes a transformation in the second post-natal week, becoming “hippocampal network oscillations” as GABA_A_ receptors become inhibitory and interneuron circuitry matures (Murata and Colonnese, 2020; Dard et al., 2022). Finally, around the third post-natal week, these immature rhythms are substituted by mature state-dependent rhythms that involve specific assemblies of neurons with complex connections and electrophysiological characteristics including mature theta and gamma oscillations and sharp-wave ripples (Egorov and Draguhn, 2013). Disruption of early activity has been linked to cognitive deficits (Krüger et al., 2012) and possibly to psychiatric disorders (Lisman et al., 2008).

Unfortunately, an assay of these activity patterns and their development *in vivo* is a time-consuming and technically sophisticated process requiring invasive recordings or imaging. This precludes screening of mouse lines or testing treatment regimes. It is important, therefore, to develop simpler methods to characterize the development of activity within the hippocampal formation. One potential marker of activity maturation is cFOS, an immediate-early gene whose expression is correlated with task engagement in multiple brain structures (Sharp et al., 1993; Hughes and Dragunow, 1995; Herdegen and Leah, 1998). cFOS expression requires combined activation of excitatory synapses (Luckman et al., 1994; Cirelli and Tononi, 2000) and intracellular signaling (Lyons and West, 2011) resulting from noradrenaline release from the locus coeruleus (Cirelli and Tononi, 2000). As a consequence, in the dentate gyrus granule cell layer, Ammon's horn pyramidal cell layer, subiculum, and entorhinal cortex of adult rodents, cFOS expression is high during wakefulness, even without specific behavioral activation, but nearly absent during sleep (Pompeiano et al., 1994; Cirelli et al., 1995).

The potential for cFOS immunohistochemistry as a marker of functional development has not been extensively explored, and its correlation with developing activity patterns and behavioral states is poorly understood. We examined sleeping and awake mice at multiple time points during development and in adulthood to ask three questions: (1) Is cFOS expression driven by early activity or is it only expressed with the onset of mature activity?; (2) At which age does cFOS expression become state-dependent?; (3) Does cFOS induction in the hippocampal formation follows a particular principle such as the progression of cell structural maturation, the direction of activity through the circuitry, or perhaps the “rhinal to dentate” neurogenetic gradient (Bayer and Altman, 1987)? To provide additional understanding of the principles of cFOS development, we expand our analysis beyond the well-characterized hippocampal and entorhinal areas to include the subicular complex and some of their major afferents and efferent areas, including retrosplenial, perirhinal, and postrhinal cortices, medial and lateral septal nuclei, supramammillary, medial and lateral mammillary nuclei, anterodorsal and anteroventral thalamic nuclei, and reuniens nucleus. Finally, we also examined the posterior hypothalamic nucleus since it projects to both the medial septal nucleus and supramammillary nucleus (all part of the theta-rhythm system). Our results suggest that cFOS staining may provide a reliable and sensitive biomarker for hippocampal formation activity development, particularly in regard to the attainment of a normal waking state, and we reveal a number of connected regions that provide an assay for early activity.

## Materials and methods

### Animals

C57BL/6 pregnant females (Hilltop Lab Animals, Scottsdale, PA) were kept in a designated room with constant temperature and humidity and a 12/12-h light/dark cycle. Pups were obtained at post-natal day (P)5, P9, P13, and P17, with P0 being the day of birth. A total of 10 mouse dams were used to bring forth all pups. Adults were also used (age range P75–P144; Ad in Tables and Figures). All experiments were completed between 1 pm and 4 pm, 6–9 h after lights on. For each age group, animals for the awake group were transferred to a new cage and kept awake for 1 h (2 h in adults) before sacrifice (*n* = 4, two males and two females for each age group; pups were handled in pairs, to decrease separation stress). To enforce wakefulness when the animals stopped walking or closed their eyes, two novel objects were separately introduced in the cage. Afterward, the animals were gently touched with a small brush when needed. The youngest animals were also kept in the palm of the hand, and when immobile they were gently touched with a finger to elicit movement. All animals of all ages in the awake group were submitted to this procedure. Animals for the sleeping group were taken directly from their home cage and immediately sacrificed (*n* = 4, two males and two females for each age group). For perfusion, animals were deeply anesthetized with an IP injection of 2,2,2,-tribromoethanol (20 mg/mL solution in sterile water containing 2.5% 2-methyl-2-butanol, used at 15 μl/g of weight; chemicals from Sigma-Aldrich, St. Louis, MO). The level of anesthesia was verified by tail pinch. The animals were transcardially perfused with PBS (VWR, Radnor, PA) containing 10 U/ml heparin (2 min) and then with fixative solution (4% paraformaldehyde in PBS; Electron Microscopy Sciences, Hatfield, PA; 10 min). A peristaltic pump (Control Company, Houston, TX) was used with a flow that was appropriate for the weight (range 0.3–7.0 mL/min).

### Tissue processing and examination

**Figure 1:**
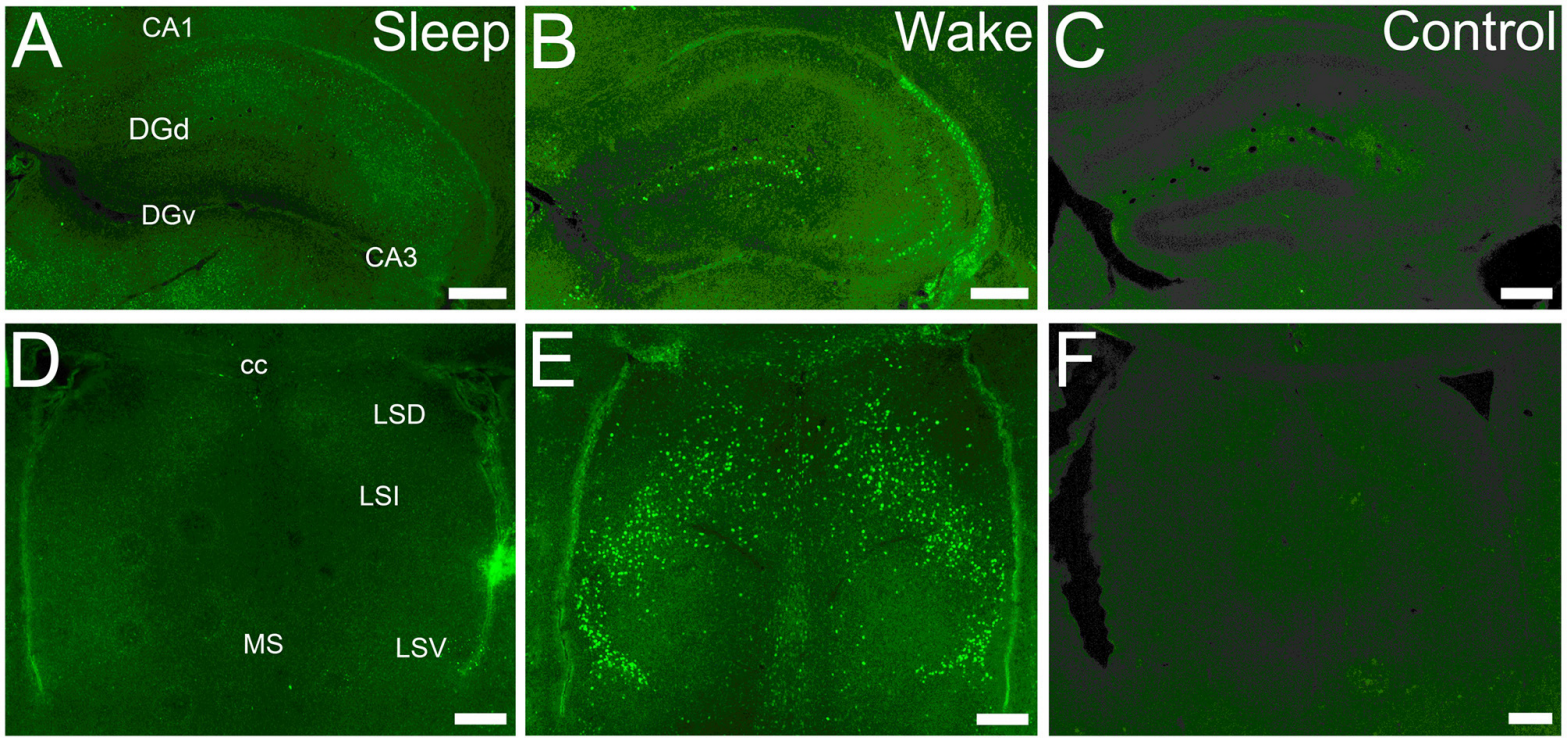
cFOS expression is state-dependent in first post-natal week. **(A)** Dorsal hippocampus at P5 in a sleeping mouse. No neurons are clearly labeled by cFOS antibody. **(B)** P5 staining in an awake animal. Clearly labeled neurons are sparsely distributed. **(C)** Negative control. No staining is seen in an awake P5 mouse in the absence of the primary antibody. **(D)** Lateral and medial septal nuclei in a P5 sleeping mouse. No neurons are labeled. **(E)** P5 staining in an awake mouse. Clearly stained neurons distributed throughout. **(F)** Negative control. No staining is detected in an awake P5 mouse. cc, corpus callosum; DGd, dentate gyrus, dorsal blade; DGv, dentate gyrus, ventral blade; LSD, LSI, LSV, dorsal, intermediate, ventral parts of the lateral septal nucleus; MS, medial septal nucleus. Scale bars: 200 μm.

After perfusion, the heads were kept in a fixative solution at 4°C for 16 h. The brains were then removed from the skull, rinsed with ice-cold PBS, and cryoprotected in 10% sucrose/PBS at 4°C for 1 day and then in 30% sucrose/PBS at 4°C for 2–3 days until they sank. Brains were frozen in embedding medium (Tissue-Tek O.C.T. Compound, Sakura Finetek USA, Inc., Torrance, CA) at −80°C and finally cut in 40 μm-thick coronal sections on a cryostat (HM505E, Microm International GmbH, Walldorf, Germany). Sections were deposited on SuperFrost glass slides (Thermo Fisher Scientific) in series of twelve and kept at −80°C until use. For each animal, one slice of every twelve was stained for cFOS following standard fluorescent immunohistochemical protocols. Smaller areas such as thalamic and mammillary nuclei were mostly present only in one stained section. Other areas were present in at least 2–3 sections. All procedures were carried out at room temperature. In brief, slides were incubated with a blocking solution containing 5% normal donkey serum (Sigma-Aldrich) and 0.5% Triton X-100 (Sigma-Aldrich) in PBS for 1 h and then with a PBS solution containing 1% normal donkey serum, 0.5% Triton X-100, and a rabbit polyclonal anti-cFOS primary antibody (ABE457, Millipore, Burlington, MA; 1:1000) overnight. After washing, the slides were incubated with an Alexa Fluor 488 anti-rabbit secondary antibody (A21206, made in donkey, Thermo Fischer Scientific; 1:200) for 2 h. Slides were counterstained with DAPI (Thermo Fisher Scientific; 0.1 μg/ml in PBS) and coverslipped with Fluoromount-G (Electron Microscopy Sciences). The omission of the primary antibody did not yield any labeling (Figures 1C, F). An identical neuroanatomical pattern of exclusively nuclear staining was obtained by using a rabbit monoclonal antibody (#2250, Cell Signaling Technology; used at 1:1000 dilution). Both antibodies are widely used in mice (see product websites). Images were acquired using a tiling microscope (DM6000B, Leica Microsystems, Inc., Exton, PA) with a 10X objective magnification and examined with Photoshop (Adobe; San Jose, CA). The number of stained cells was quantified blind to age and condition since the images were coded. Images were imported in TIFF format into open-source ImageJ software (Rasband, W.S., ImageJ, U. S. National Institutes of Health, Bethesda, Maryland, USA, https://imagej.nih.gov/ij/) where they were converted into 8-bit grayscale images. Regions of interest were outlined, and their area was measured. cFOS-labeled cells were counted in outlined areas using the Particle Analyzer tool, and their density was expressed in a number of cells/mm^2^. In the case of a bilateral structure, the value for that area was calculated as the mean density between the two sides for each animal. The mean staining levels reported in Table 1 (number of cells/mm^2^ ± SE) were calculated based on the observations from four animals, with a few exceptions as noted. No difference was evident between the two male and two female animals of each group. Brain areas were identified following developing and adult mouse brain atlases (Paxinos et al., 2007; Paxinos and Franklin, 2019).

**Table 1:** cFOS expression (in labeled cells/mm^2^ ± SE) in waking and sleeping mice of different ages.

**Brain areas**	**P5**	**P9**	**P13**	**P17**	**Ad**	***p***	**>P5**	**=Ad**
**WAKING**								
**Hippocampus**								
CA1, Pyramidal cell layer	96 ± 52	399 ± 157	1045 ± 309	1901 ± 138	3283 ± 525	<10^−5^	P17	Ad
CA3, Pyramidal cell layer	170 ± 46	465 ± 113	669 ± 73	743 ± 88	940 ± 145	0.00074	P13	P13
DG, Granule cell layer^**d**^	**268** **±73**	633 ± 61	536 ± 43	493 ± 95	551 ± 83	0.03306	P9	P9
DG, Granule cell layer^**v**^	7 ± 7	153 ± 41	245 ± 65	299 ± 32	321 ± 71	0.00244	P13	P9
**Entorhinal cortex**								
EC Layer(L)2	**563** **±67**	630 ± 89	706 ± 113	610 ± 59	1025 ± 294	0.25469	ns	ns
EC L3	67 ± 22	251 ± 62	369 ± 31	489 ± 114	659 ± 115	0.0011	P17	P17
EC L5b	35 ± 6	93 ± 19	169 ± 13	270 ± 64	267 ± 51	0.00153	P17	P13
EC L6	**115** **±23**	315 ± 42	398 ± 64	434 ± 104	362 ± 54	0.02306	P13	P9
**Subicular complex**								
Dorsal Subiculum	103 ± 22	124 ± 7	186 ± 36	191 ± 53	756 ± 100	<10^−5^	Ad	Ad
Ventral Subiculum	**220** **±36**	847 ± 131	884 ± 117	757 ± 149	822 ± 94	0.0043	P9	P9
Postsubiculum, L2-3	47 ± 5	338 ± 74	1247 ± 361	1812 ± 242	2649 ± 122	<10^−5^	P13	Ad
Postsubiculum, L4-6	**232** **±35**	265 ± 20	166 ± 39	1269 ± 348	1684 ± 133	<10^−5^	P17	P17
**Septal Nuclei (SN)**								
Lateral SN, Dorsal	**64** **±19**	84 ± 30	169 ± 60	114 ± 16	110 ± 35	0.34846	ns	ns
Lateral SN, Intermediate	**482** **±140**	594 ± 130	701 ± 111	576 ± 49	697 ± 185	0.74167	ns	ns
Lateral SN, Ventral	**1091** **±162**	881 ± 183	939 ± 217	793 ± 132	802 ± 218	0.78273	ns	ns
Medial SN	**125** **±40**	115 ± 15	190 ± 53	159 ± 40	252 ± 22	0.11063	ns	ns
**Other Areas**								
Anterodorsal Thalamic N	**792** **±169**	298 ± 38^∧^	230 ± 106	26 ± 7	44 ± 29	0.00033	P9^*^	P9
Anteroventral Thalamic N	**55** **±10**	27 ± 5^∧^	38 ± 29	157 ± 45	227 ± 74	0.0225	Ad	P17
Reuniens Nucleus	53 ± 11	65 ± 12	91 ± 12	139 ± 30	763 ± 148	<10^−5^	Ad	Ad
Posterior Hypothalamic N	**266** **±74**	278 ± 20	616 ± 80	821 ± 115	928 ± 120	0.00016	P13	P13
Supramammillary N	**517** **±54**^**∧**^	366 ± 41	534 ± 55	1104 ± 245	1068 ± 119	0.00324	P17	P17
Retrosplenial Cortex	33 ± 2	35 ± 5	268 ± 72	908 ± 127	1112 ± 201	<10^−5^	P17	P17
Perirhinal Cortex	43 ± 7	122 ± 19	353 ± 43	600 ± 80	826 ± 70	<10^−5^	P13	Ad
Postrhinal N	21 ± 8	63 ± 16	247 ± 113	261 ± 42	356 ± 21	0.00285	P13	P13
**SLEEPING**								
**Hippocampus**								
CA1, Pyramidal cell layer	17 ± 10	7 ± 5	22 ± 8	6 ± 6	0 ± 0	0.17853	ns	ns
CA3, Pyramidal cell layer	12 ± 9	16 ± 11	40 ± 6	46 ± 9	9 ± 6	0.02041	P17	Ad
DG, Granule cell layer^**d**^	0 ± 0	35 ± 15	166 ± 15	192 ± 11	73 ± 29	<10^−5^	P13	Ad
DG, Granule cell layer^**v**^	35 ± 35	21 ± 15	55 ± 22	149 ± 34	75 ± 31	0.04883	P17	ns
**Other Areas**								
Medial SN	2 ± 2	7 ± 5	8 ± 3	11 ± 8	5 ± 3	0.64817	ns	ns
Anterodorsal Thalamic N	110 ± 45	37 ± 21	20 ± 14	0 ± 0	0 ± 0	0.02249	P13^*^	P9
Anteroventral Thalamic N	11 ± 4	0 ± 0	0 ± 0	1 ± 1	1 ± 1	0.00433	P9^*^	P9

The last three columns show the one-dimension ANOVA for the effect of age. For each area, “p” indicates the statistical significance for age effect, “>P5” indicates the age at which the cFOS expression values are first higher than at P5, and “=Ad” indicates at which age they reach adult levels, both measured by post hoc difference. Bold numbers in the P5 column indicate significant expression at P5. All groups n = 4 except ^**∧**^n = 3. ^*^Decrease in cell density. ^**v**^ ventral blade, ^**d**^ dorsal blade, ns non-significant effect of age.

### Statistical analysis

Development of cFOS-label density (Table 1) was quantified by one-factor ANOVA for each region, followed by *post hoc* (Dunnett) test for significant differences for each age group between P5 and adult groups (*p* < 0.05). To test for significant staining at P5 (Table 1), a one-sample one-tailed *t*-test for an increase from 0 was used for each region. The resulting p-value was adjusted for multiple comparison by the Holm step-down method. For cluster analysis (Figure 10), cell densities were normalized to the mean density for that region for all ages and the normalized time series was subjected to hierarchical clustering based on centroid distance (Matlab function ‘linkage'; MathWorks, Natick, MA). For display, the order of the dendrogram and intensity display were manually ordered by time developmental delay without crossing dendrogram lines.

## Results

### Quantitative analysis of cFOS staining during wake and sleep

We examined cFOS expression in two groups: an enforced waking (“Waking”) group for which the animals were maintained in a waking state by manual manipulation before sacrifice, and a group sacrificed immediately after removal from their homecage during the light-on period while asleep (“Sleeping” group). cFOS expression was strongly state-dependent already by P5 (Figures 1A, B, D, E). Values obtained from the two groups are reported in Table 1. In the sleeping group, cFOS levels were very low in almost all regions examined at all ages (Figures 1A, D). A prominent exception to this was the dentate gyrus which had significant expression during sleep at P13 and P17 that decreased in adults. In addition, the anterodorsal thalamic nucleus was consistently stained at P5 but not at later ages. Because cFOS staining was so low and limited in the sleeping group, we restricted our quantification of cFOS reactivity during sleep to a few regions of interest shown in Table 1. A one-sample *t*-test did not reveal any region with cFOS expression that was significantly greater than zero at P5. ANOVA analysis for the effect of age showed only DG and CA3 to significantly increase cFOS expression with age, peaking at P17. The anterodorsal and anteroventral nuclei significantly reduced cFOS expression with age in sleeping animals.

**Figure 2:**
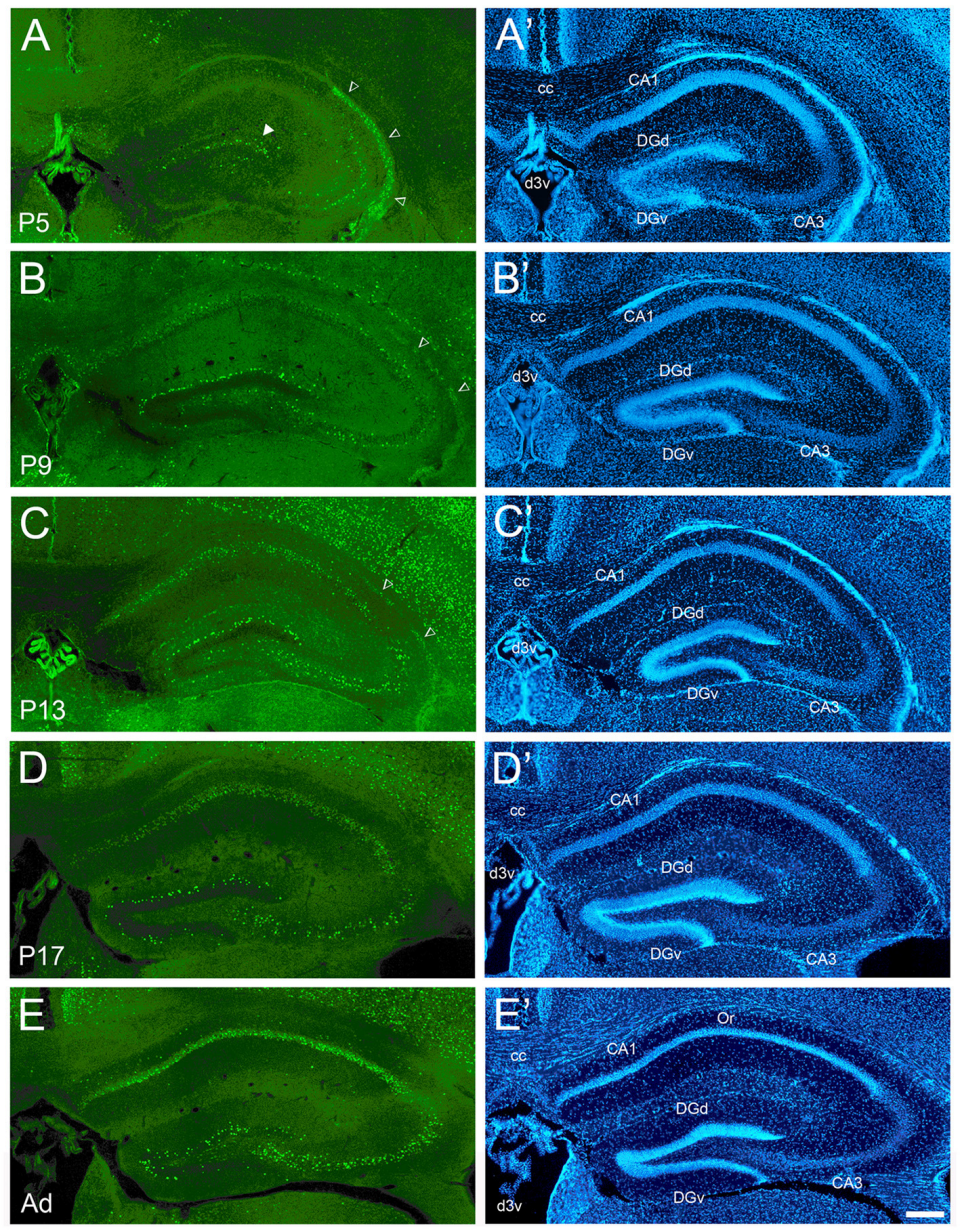
Development of cFOS expression in the dorsal hippocampus of awake mice. **(A)** At P5, consistent staining is observed in the dorsal blade of the dentate gyrus granule cell layer (DGd), especially in its lateral part (full arrowhead). CA3 expression was observed in this animal and was only scattered in others. Dense staining of the neuroepithelium (empty arrowheads) was observed at P5 and decreased through P13. **(B)** At P9, staining density increased in DGd and extended to the ventral blade (DGv). Scattered but consistent staining was observed in the pyramidal cell layer of CA1 and CA3 in all pups. **(C, D)** At P13 and P17, staining increased in DGv and CA3. **(E)** Adult animals showed strongly increased staining in CA1 pyramidal layer and increased but still low staining in the oriens layer of CA1 (Or). **(A'–E')** DAPI counterstaining of the same coronal sections shown in **(A–E)**. cc, corpus callosum; d3v, dorsal 3rd ventricle. Scale bar: 200 μm (all panels).

**Figure 3:**
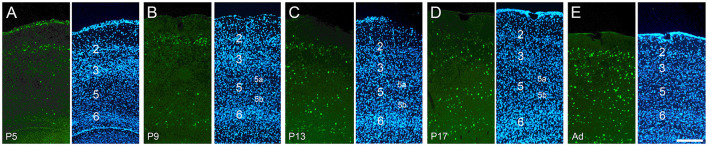
Development of cFOS expression in the entorhinal cortex of awake animals. Coronal sections of the lateral entorhinal cortex, at intermediate anteroposterior levels. **(A)** At P5, consistent staining was seen only in L2, with scattered labeling in other layers. **(B)** At P9, consistent L6 staining and scattered L5b staining emerged. **(C, D)** All layers except L5a have adult levels of staining by P13 and P17. **(E)** At adulthood, substantial levels of staining were seen in all layers, including L5a **(A'–E')**. DAPI counterstaining of the same coronal sections shown in **(A–E)**. Scale bar: 200 μm (all panels).

**Figure 4:**
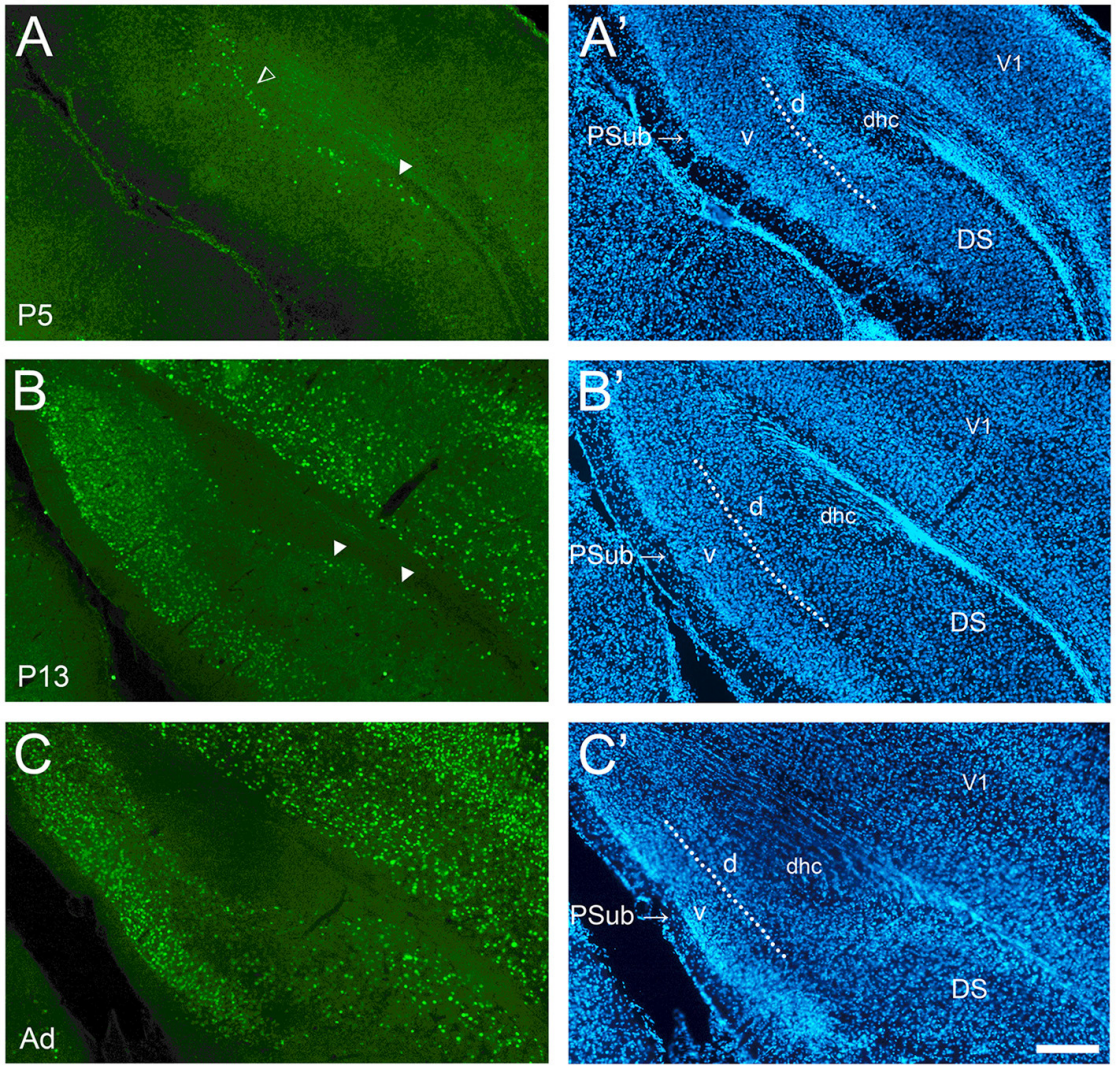
Development of cFOS in the dorsal subiculum and postsubiculum of awake animals. **(A)** At P5, scattered stained cells were observed only in the deepest part of the pyramidal cell layer of the dorsal subiculum (DS) (full arrowheads) and in the dorsal lamina of the postsubiculum (PSub) (open arrowhead). **(B)** At P13, staining remains scattered in deep layers of DS but becomes widespread in PSub. **(C)** By adulthood, the staining in the DS increased to include lightly and moderately stained cells throughout the pyramidal layer. Staining increased to moderate levels throughout the PSub in adult animals **(A'–C')**. DAPI counterstaining of the same coronal sections shown in **(A–C)**. The dotted lines in PSub indicate the separation between dorsal lamina (L2-3; d) and ventral lamina (L4-6; v). dhc, dorsal hippocampal commissure; V1, primary visual cortex. Scale bar: 200 μm (all panels).

**Figure 5:**
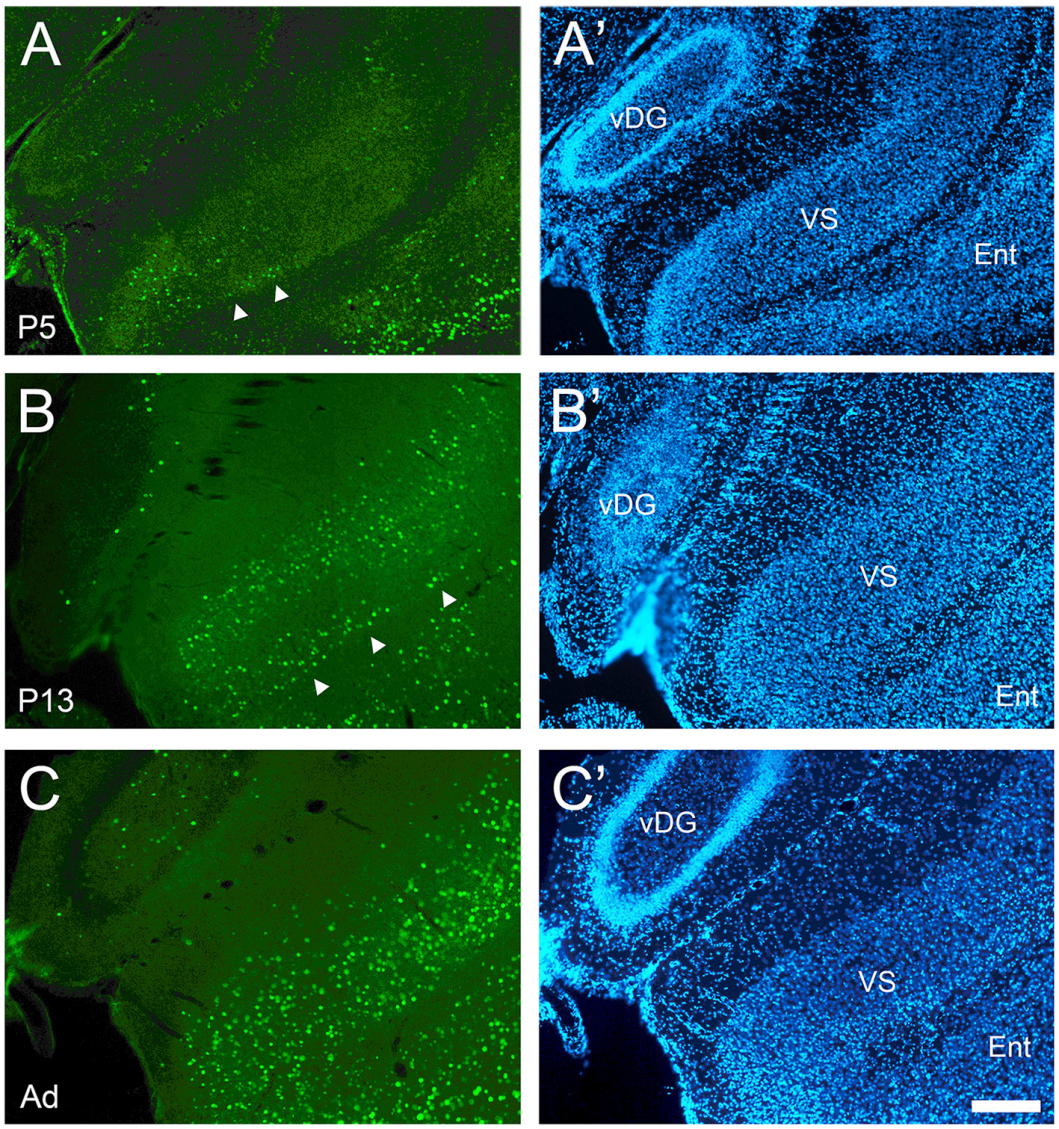
Development of cFOS expression in the ventral subiculum of awake animals. **(A)** At P5, cFOS-stained cells were first detected in very small numbers in the deepest part of the pyramidal cell layer of the ventral subiculum (VS) (full arrowheads). **(B)** By P13, cFOS expression increased to adult levels in terms of cell densities in the pyramidal cell layer with stained cells both in the deep part (arrowheads) and the more superficial part, which were separated by an area lacking staining. Cells were stained at low intensities. **(C)** By adulthood, the cells were strongly labeled and homogeneously distributed throughout the depth of the VS pyramidal cell layer. **(A'–C')** DAPI counterstaining of the same coronal sections shown in **(A–C)**. Ent, entorhinal cortex; vDG, ventral dentate gyrus. Scale bar: 200 μm (all panels).

**Figure 6:**
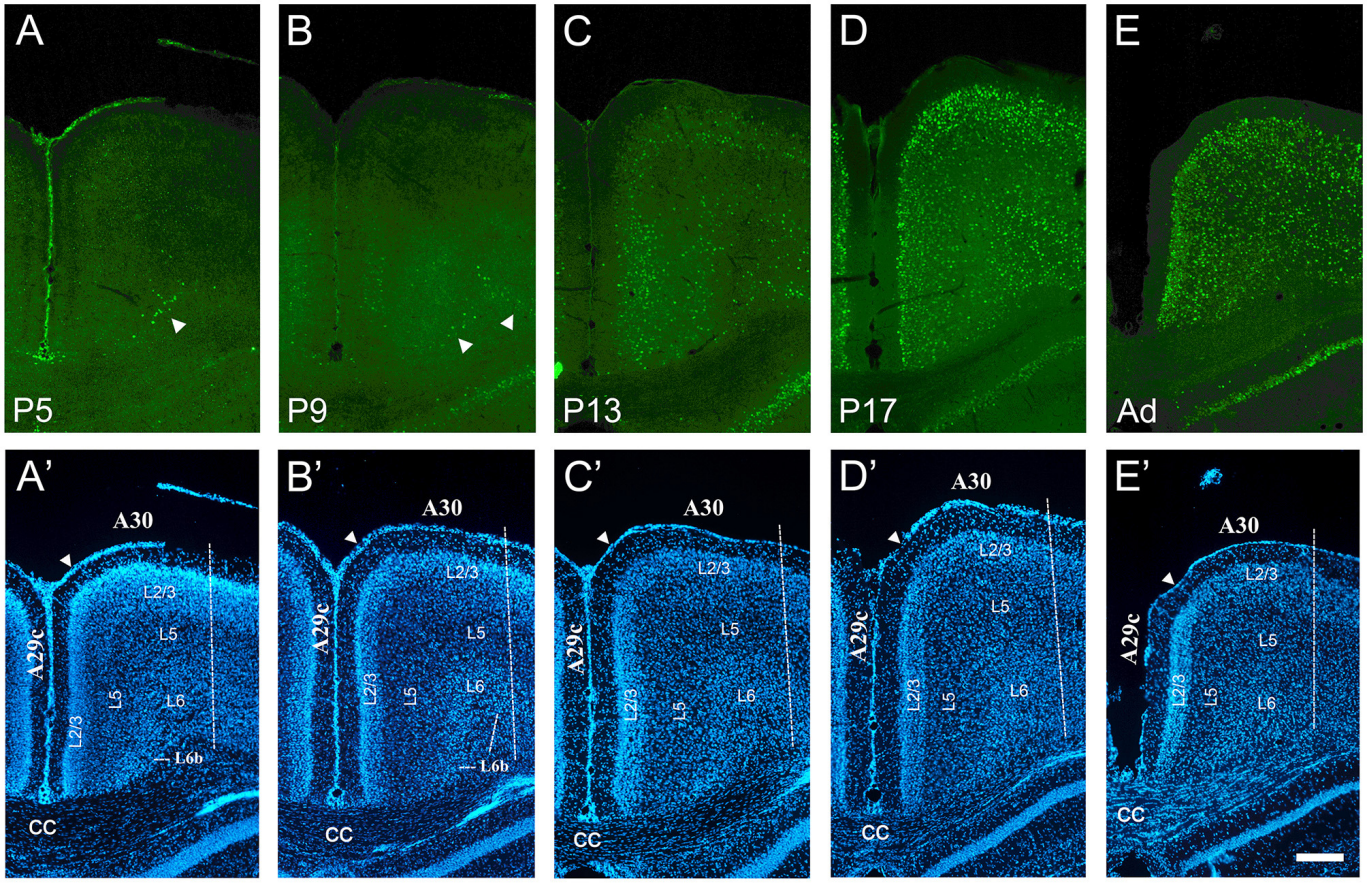
Developmental increase of cFOS in the retrosplenial cortex of awake animals. **(A)** At P5, stained cells were detectable only in L6b of the retrosplenial granular area 29c (A29c; arrowhead). **(B)** At P9, staining began in L3 and L5b and remained in L6b of A29c. Staining in L6b of retrosplenial dysgranular area 30 (A30) was first observed at this age. Two arrowheads point to L6b of A29c and A30. **(C)** By P13, low cFOS levels were seen in L3, L5b, and L6b of both A29c and A30, while L2 was clearly stained only in A29c. **(D, E)** Staining was moderate in all layers of both A29c and A30 by P17 and adulthood, respectively. **(A'–E')** DAPI counterstaining of the same coronal sections shown in **(A–E)**. The white arrowheads in A'–E' indicate the approximate superficial boundary between A29c and A30, while the dotted line indicates the approximate lateral boundary of A30. cc, corpus callosum. Scale bar: 200 μm (all panels).

**Figure 7:**
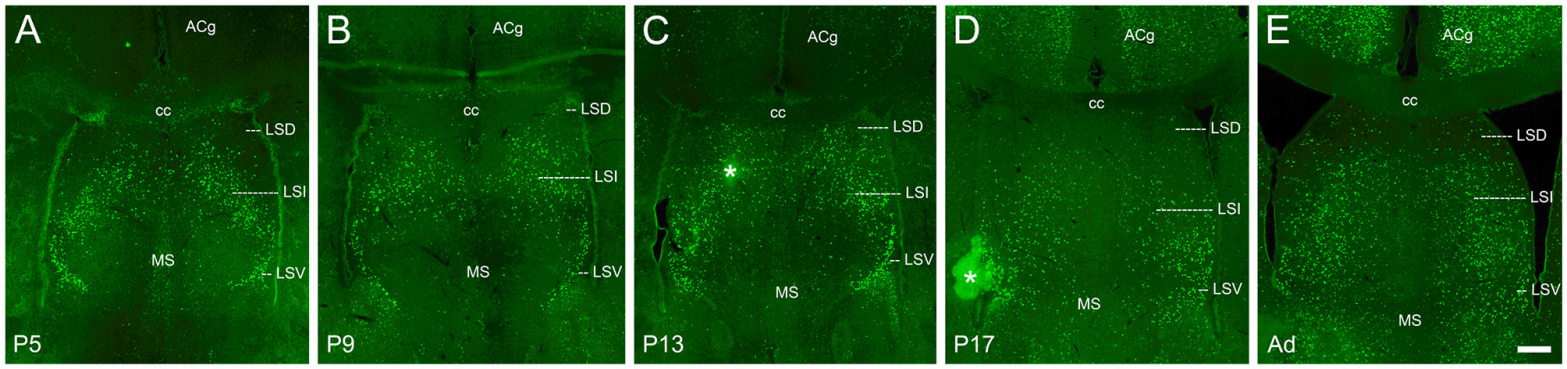
cFOS expression in the septum of awake animals. **(A)** By P5, consistent dense staining was observed in both the intermediate (LSI) and ventral (LSV) parts of the lateral septal nucleus. Occasional cells were observed in the dorsal part (LSD). **(B–E)** cFOS levels remained intermediate in the LSI and LSV and very low in the LSD at P9, P13, P17, and adulthood, respectively. Staining in the medial septal nucleus (MS) was low at all ages. The asterisks indicate artifacts. ACg, anterior cingulate cortex; cc, corpus callosum. Scale bar: 200 μm (all panels).

**Figure 8:**
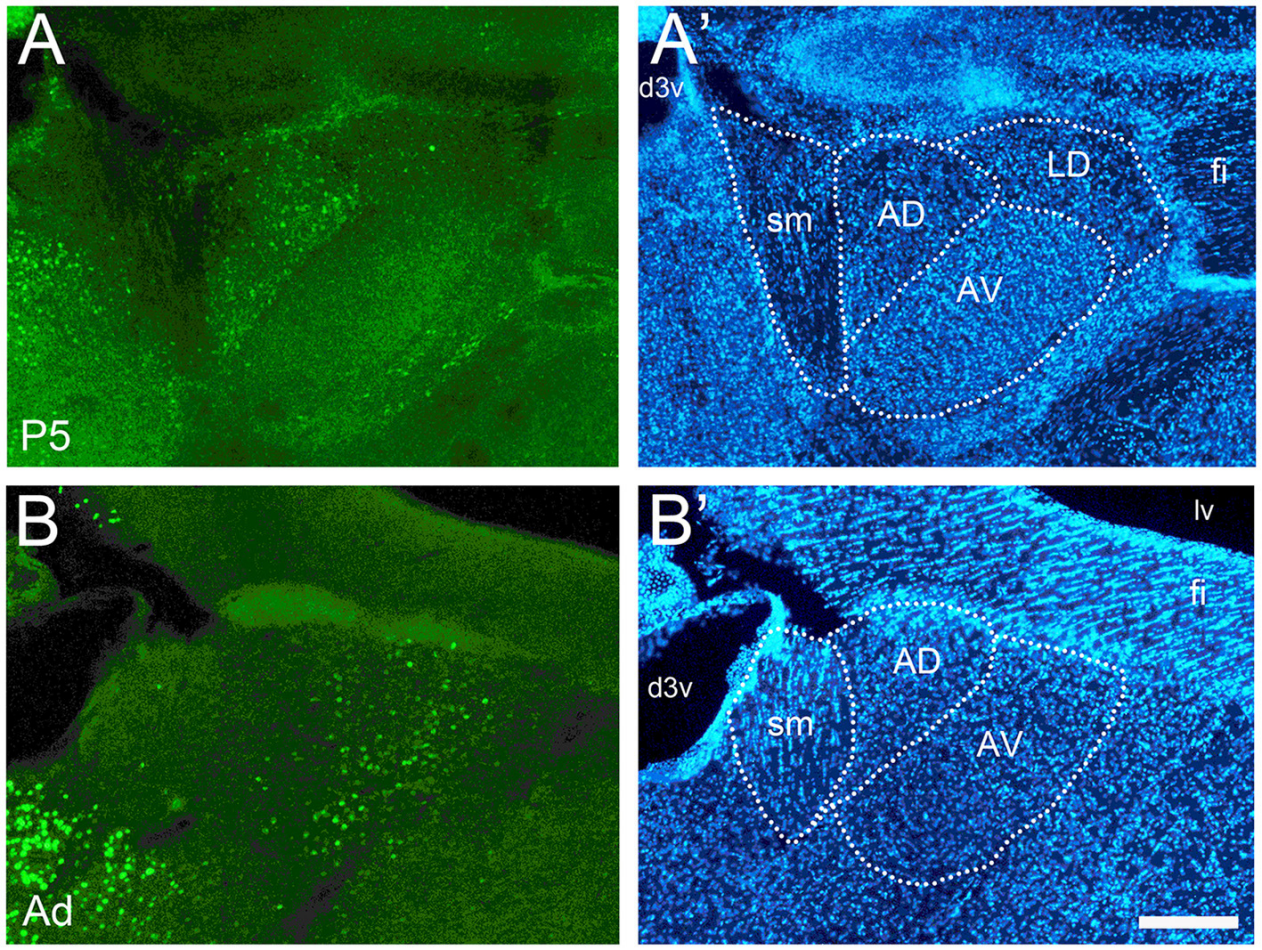
Developmental changes of cFOS expression in anterior thalamic nuclei of awake animals. **(A)** At P5, cFOS staining was seen in the anterodorsal nucleus (AD) but not in the anteroventral nucleus (AV). **(B)** Adults, however, showed the inverse pattern with increased staining in AV but very little in AD. **(A'** and **B')** DAPI counterstaining of the same coronal sections shown in A and B. d3v, dorsal 3rd ventricle; fi, fimbria; LD, laterodorsal thalamic nucleus; lv, lateral ventricle; sm, stria medullaris. Scale bar: 200 μm (all panels).

**Figure 9:**
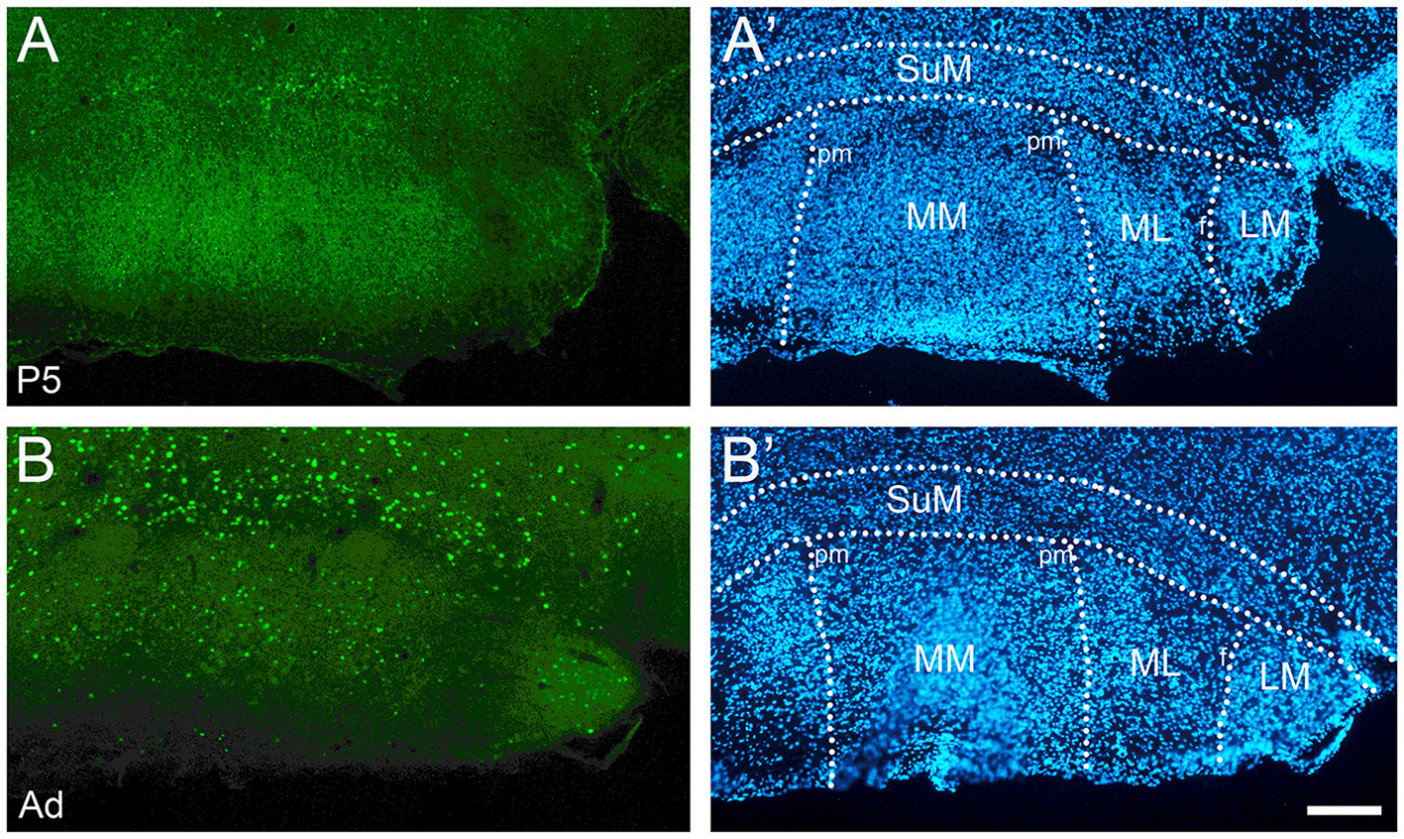
Developmental increase of cFOS expression in the mammillary body of awake animals. **(A)** P5 pups showed low levels of cFOS limited to the supramammillary nucleus (SuM). **(B)** In adults, cFOS increased to intermediate levels in the SuM and to low levels in the medial and lateral parts of the medial mammillary nucleus (MM and ML) and the lateral mammillary nucleus (LM). **(A'** and **B')** DAPI counterstaining of the same coronal sections shown in **(A, B)**. pm, principal mammillary tract. Scale bar: 200 μm (all panels).

**Figure 10:**
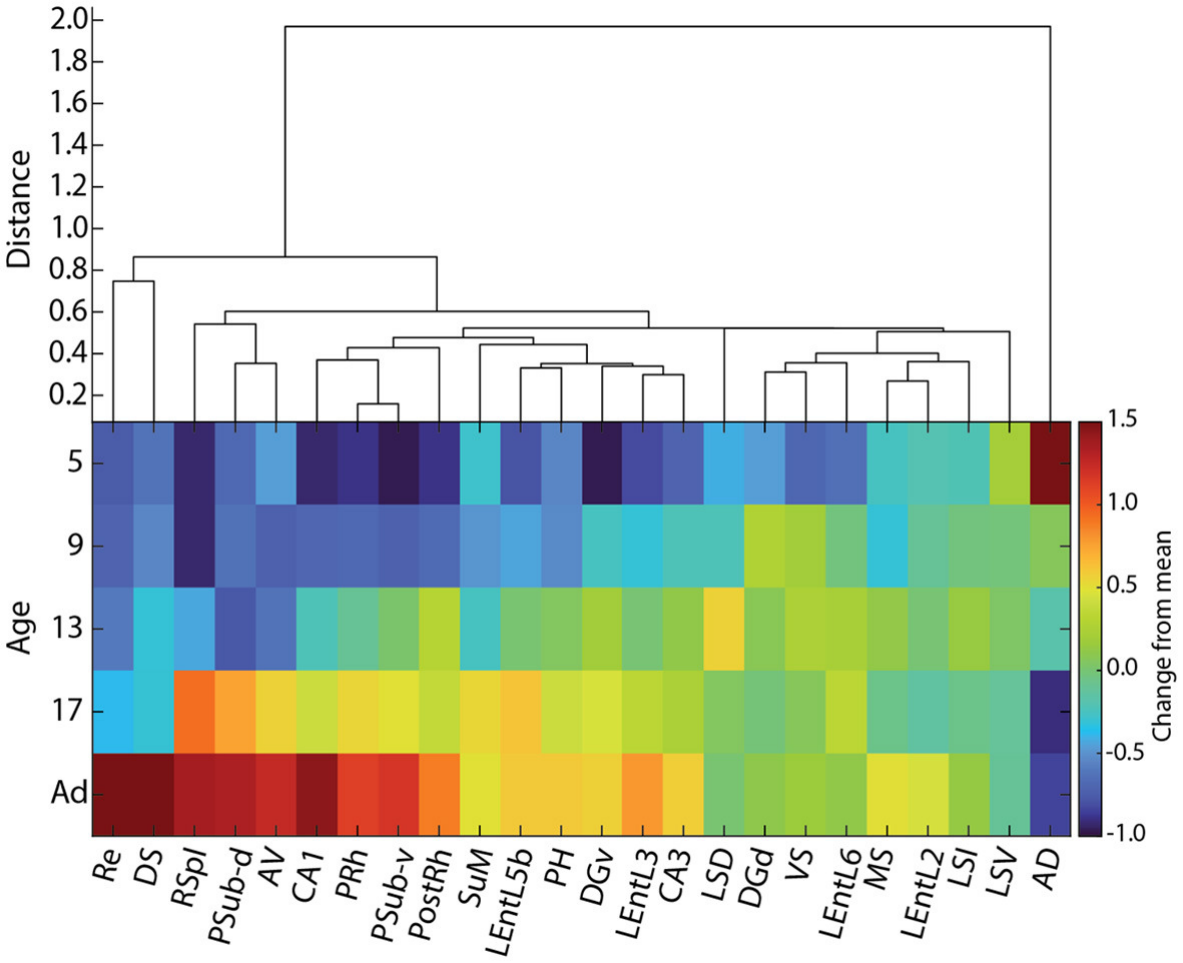
Hierarchical clustering of cFOS developmental patterns in awake animals. **(Top)** Hierarchical clustering of cFOS expression normalized to adult levels. **(Bottom)** Normalized cFOS expression by age and region. Colormap is the log ratio of cFOS expression at each age relative to adult (Ad) levels ordered according to clustering and the time that cFOS expression reaches adult levels. AD, anterodorsal thalamic nucleus; AV, anteroventral thalamic nucleus; DGd, dentate gyrus, dorsal blade; DGv, dentate gyrus, ventral blade; DS, dorsal subiculum; LEntL2, -L3, -L5b, -L6, layer 2, 3, 5b, 6 of the lateral entorhinal cortex; LSD, LSI, LSV, dorsal, intermediate, ventral parts of the lateral septal nucleus; MS, medial septal nucleus; PH, posterior hypothalamic nucleus; PostRh, postrhinal cortex; PRh, perirhinal cortex; PSub-d, postsubiculum, dorsal lamina (L4-6); PSub-v, postsubiculum, ventral lamina (L2-3); Re, reuniens nucleus; RSpl, retrosplenial cortex; SuM, supramammillary nucleus; VS, ventral subiculum.

At every age and every region examined, cFOS expression was greater in the waking group and so this group was subjected to more extensive qualitative and quantitative analyses (Table 1; Figures 2–10). For many regions examined in the extended hippocampal axis, staining was absent or low in the first post-natal week (P5). The anterodorsal and anteroventral thalamic nuclei, lateral and medial septal nuclei, L2 and L6 of the entorhinal cortex, dentate gyrus dorsal blade ventral subiculum, postsubiculum dorsal layers (L4-6), and posterior hypothalamic and supramammilary nuclei had expression levels that were significantly greater than zero and more than 20% of adult levels. Most of these regions, as well as all of the other regions examined, increased their expression with age. Two regions, the ventral part of the lateral septal nucleus and anterodorsal thalamic nucleus, expressed cFOS at very high densities at P5, and this expression either remained high-throughout development (septum) or actually decreased (anterodorsal) during development.

Hierarchical clustering of normalized cFOS expression levels (Figure 10) revealed multiple patterns of developmental change in cFOS expression. Most interestingly, examined areas did not cluster by brain region, but rather each region appears to have early and late expressing areas. The anterodorsal thalamic nucleus was in a distant cluster, reflecting the fact that it was the only region to decrease staining during development. The reuniens nucleus and dorsal subiculum constituted the next most isolated cluster, showing strong increases in development that only reached high expression levels as adults. The next latest-developing cluster contained the retrosplenial cortex, postsubiculum dorsal layers (L4-6), and anteroventral thalamic nucleus. This group was preceded in development by a cluster containing CA1 of the hippocampus, postsubiculum ventral layers (L2-3), and perirhinal and postrhinal cortices. The dorsal and ventral parts of the lateral septal nucleus formed notable isolated branches among the cluster of early developing regions. Altogether, our results show that the maturation of cFOS reactivity reflects the specific functional development of individual components of nearby regions, rather than a more general gradient or regional factor.

A detailed qualitative description of the developmental expression patterns in each region is presented below.

### Hippocampus

Overall, the maturation of cFOS expression followed the pattern of the classic hippocampal tripartite circuit: DG:CA3:CA1. Similar timing was seen in both the dorsal and ventral hippocampus, in agreement with recent observations (Signature 2 in Figure 1B and Supplementary material 1 in Olsen et al., 2023) (Table 1 shows values for the dorsal hippocampus). The first region in the hippocampus to express cFOS was the dorsal blade of the dentate gyrus granule cell layer, which had reliable though less than adult levels of staining at P5 (Figure 2A). Cells showed strong staining, and their density was higher in the lateral part of the blade. Expression increased to adult levels in the dorsal blade by P9 (intermediate levels; Figure 2B) and in the ventral blade by P13 (low levels; Figure 2C). Until P17, labeled cells were clustered at the border between the granule cell layer and the molecular layer (Figures 2A–D) and only became distributed throughout the whole granule cell layer in adult animals, in both blades (Figure 2E).

In Ammon's horn, very low staining was seen with just a few stained cells at P5 (CA1 in Figure 2A). One of the P5 animals showed stronger staining in CA3 (low levels; Figure 2A). A low density of strongly stained cells was consistently observed in the pyramidal cell layer of both CA1 and CA3 by P9 (Figure 2B). Adult levels of staining were reached in CA3 on P13 (Figures 2C–E) but in the CA1 pyramidal layer only after P17 (Figure 2E). While generally restricted to the pyramidal cell layers at all ages, a low amount of cFOS expression was reliably detected in the CA1 oriens layer in adult animals (Figure 2E). Strong staining was also seen in what appears to be part of the Ammon's horn neuroepithelium at P5 (Figure 2A; empty arrowheads) (Paxinos et al., 2007) which decreased to low levels at P9 and P13 (Figures 2B, C; empty arrowheads) and was not detectable at older ages.

### Entorhinal cortex

Overall cFOS staining in the entorhinal cortex was relatively high at P5 and increased throughout development, looking mature by P17. There were significant differences, however, between layers. The earliest cFOS expression was observed in layer (L) 2 at P5 (Figure 3A), when it was already adult-like (Figure 3E). L3 staining was absent at P5 and limited to its most posterior levels on P9 which spread to all anteroposterior levels by P13 (Figure 3C) and increased to adult levels on P17 (Figures 3D, E). L5 showed only a few cFOS-stained cells at P9 (Figure 3B) and low staining at P13 (Figure 3C) and P17 (Figure 3D). Stained cells were limited to the deeper half of the layer (L5b). Staining in the whole extent of L5 was seen only in adult animals (low levels; Figure 3E). Low staining was seen in L6 starting from P9 until adulthood (Figures 3B–E). Thus, staining in the entorhinal cortex begins and matures faster in the output layers of the hippocampus (especially L2), with the input layers following along similar timelines to the output (CA1) region of the hippocampus.

### Subicular complex

The dorsal subiculum was very late developing. While it showed cFOS-positive cells in the deepest part of the pyramidal cell layer in the more posterior and distal levels at P5 and older (Figures 4A, B), only in adults did the mature pattern of a low density of lightly and moderately stained cells throughout the whole thickness of the pyramidal cell layer emerge (Figure 4C). The expression in the ventral subiculum was low at P5, with lightly stained cells detected in the deepest part of the pyramidal cell layer (Figure 5A). Adult densities of label cells were observed starting at P9 though these had low intensity (Figure 5B) which increased in adults (Figure 5C). Thus, the subiculum contained one of the first and last developing regions.

Development of cFOS expression in the postsubiculum was similar to the ventral subiculum. Low staining was first seen in its dorsal/deep/internal lamina (L4-6) starting from P5 (Figures 4A, B), and it became moderate at P17 and adulthood (Figure 4C). A few stained cells were detected in the ventral/superficial/external lamina (L2-3) at P5 (Figure 4A), and they were consistently observed at P9. Staining extended to the whole depth of both laminas of the postsubiculum by P13 (Figure 4B). In adults, the staining increased to intermediate levels in the external lamina (Figure 4C).

### Retrosplenial, perirhinal, and postrhinal cortices

The retrosplenial cortex was examined in granular area 29c and dysgranular area 30. cFOS expression began earlier in area 29c than 30, but it showed a similar progression through the layers: L6b → L3 & L5b → L2 → L5a & L6a. A low density of stained cells was first detected in L6b of area 29c at P5 and of area 30 at P9 (Figures 6A, B). Staining was first observed in the deep part of L3, L5b, and L6b in area 29c at P9 (Figure 6B) but at P13 in area 30 (Figure 6C). Area 29c showed low staining at P13, with labeled cells in L3, L5b, and L6b (Figure 6C). This staining became robust if still scattered in both regions on P13, and the density increased at P17 to adult levels. At these two latter ages, staining spanned all layers in both areas 29c and 30, and strongly labeled cells were observed in L2 (Figures 6D, E).

Similarly, in the perirhinal cortex, a few cells were seen in L6 at P5 and in L2-3 and L6 at P9. Consistent staining, now including L5b, was observed at P13. All layers were stained at low and intermediate levels by P17 and adulthood, respectively. At these two latter ages, staining was stronger in the dorsal than the ventral part of the perirhinal cortex (corresponding to areas 36 and 35, respectively). The postrhinal cortex showed a similar progression of activation across its layers, with cFOS expression seen in L5a only after P17.

### Septum

The septum was remarkable as all of the subdivisions we examined here showed significant cFOS expression at P5 and this expression remained relatively constant at all ages. While the expression in the medial septal nucleus and the dorsal part of the lateral septal nucleus was low but consistent at all ages (Figures 7A–E), the intermediate and ventral parts of the lateral septal nucleus maintained prominent expression at all ages (Figures 7A–E).

### Thalamic and hypothalamic areas

The anterodorsal thalamic nucleus, like the lateral septum, was unusual in that it was consistently stained at P5 (Figure 8A) but had almost no staining thereafter (Figure 8B). The anteroventral thalamic nucleus and the reuniens nucleus showed very low or no staining from P5 to P17 (Figure 8A) and low levels in adults (Figure 8B).

The posterior hypothalamic and supramammillary nuclei were among the few nuclei consistently stained at P5 and P9, albeit at low levels (Figure 9A); however, unlike the anterodorsal thalamic nucleus and lateral septum staining levels increased to intermediate levels at older ages (Figure 9B), cFOS expression in the lateral and medial mammillary nuclei was absent at P5-P13 (Figure 9A), very low at P17, and low in adults (Figure 9B).

## Discussion

We evaluated the use of cFOS expression as a biomarker for the maturation of network activity in the developing hippocampus and its extended network. Immunostaining for cFOS has been a useful tool to map task-specific circuit activation, for example, in hippocampal and parahippocampal areas during object and place experiences (Vann et al., 2000; Kinnavane et al., 2015; Minatohara et al., 2016; Cinalli et al., 2020). cFOS immunohistochemistry has also been used to map sensory and motor pathways (e.g., Wan et al., 1992; Nomura et al., 2002; Illig and Haberly, 2003). A baseline level of cFOS expression is observed in a characteristic pattern, even in the absence of a specific task or sensory stimulation, likely reflecting the ongoing background activity during wakefulness as well as task-specific circuits engaged spontaneously in the homecage just before sacrifice (Zangenehpour and Chaudhuri, 2002). Therefore, we hypothesize that it may serve as a simple and convenient assay for activity development even in the absence of behavioral tasks. The hippocampus and entorhinal cortex are excellent regions for this analysis as their functional development has been intensively studied (Cossart and Khazipov, 2022; Del Rio-Bermudez and Blumberg, 2022), giving us a reference to determine the activity changes correlated with cFOS expression during development. Demonstrating such correlation is necessary as cFOS is not expressed in some regions despite robust neuronal activation (Cirelli and Tononi, 2000; Hudson, 2018).

We found that throughout the areas examined, at all ages cFOS expression was much higher following enforced wakefulness than when the animals were taken from their home cage during the lights-on period. Sleep expression was often not observed at all, even in regions with very high expressions during wakefulness. Waking expression in the hippocampus and entorhinal cortex was generally low at P5 and P9, showing that early activity bursts are not sufficient to drive cFOS expression despite their massive and synchronous activation of the network (Cossart and Khazipov, 2022; Leprince et al., 2023). The expression in these and the associated regions progressively increased in the third post-natal week, roughly following the flow of information as defined by the classical hippocampal–entorhinal circuit (e.g., Amaral and Lavenex, 2007). This pattern indicates that robust, adult-like cFOS expression in these regions is correlated with the onset of adult-like continuous activity patterns such as the theta and gamma rhythms. It also agrees with the recently described pattern of cFos developmental transcription in rat hippocampal homogenates (Signature 2 in Figure 1B and Supplementary material 1 in Olsen et al., 2023). We identified a few regions that did not follow this basic pattern of increased expression during development. Anterodorsal thalamic nucleus showed an inverse pattern, with robust early expression, but decreased adult expression. Entorhinal L2 and septum showed adult-like expression starting from P5. In total, our results suggest that cFOS immunohistochemistry can be a useful biomarker for the maturation of activity in the extended hippocampal circuitry during wakefulness.

### Comparison with previous studies on developmental and state-dependent cFOS expression

Two studies mapped cFOS expression in the brain of developing rodents at time points similar to ours (Smeyne et al., 1992; Alcantara and Greenough, 1993). In both, neither the behavioral state of the animals nor the time of the day at sacrifice were reported. The very low levels of cFOS observed in most areas even in adult animals suggest that the animals in these studies were largely sacrificed during sleep. Transient expression of cFOS was observed during development in the entorhinal L2 of rat pups (Alcantara and Greenough, 1993) and in retrosplenial cortex, CA1, CA3, septum, and anterior thalamic nuclei of mouse pups (Smeyne et al., 1992), similar to what we observed here in awake, but not sleeping pups. Because sleep in pups is more disturbed and less consolidated, the apparent transient expression observed in these studies may have been a result of poor control of the state.

We observed very low or absent staining in sleeping animals at all ages, in agreement with previous studies on adult rats (Grassi-Zucconi et al., 1993; Pompeiano et al., 1994; Cirelli et al., 1995). We observed no ‘sleep-on' regions; any expression observed during sleep was lower than in waking and largely occurred in areas showing the highest cFOS levels during wakefulness. We believe that this expression is due to interrupted sleep as pups are constantly jostled by their littermates even during sleep times. Regions such as anterodorsal thalamic nucleus, dentate gyrus, and postsubiculum which express cFOS at significant levels at P5 and P9 did so only during wakefulness, suggesting that some components of adult-like sleep/wake state regulation are intact even at these early ages, despite the poor regulation of cortical background activity (Seelke and Blumberg, 2010). One component of this intact state regulation may be the noradrenergic locus coeruleus, in which we observe high cFOS expression in waking but not sleeping animals even at the youngest ages (Pompeiano and Colonnese, unpublished data), in agreement with observations in adults (Tononi et al., 1994; Léger et al., 2009). Thus, ascending neuromodulatory inputs may provide a similar gating of cFOS expression in early post-natal development as in the adult (Cirelli et al., 1996; Cirelli and Tononi, 2004).

It is unclear the degree to which the increased cFOS expression observed in the forced wakefulness state is a result of extended wakefulness *per se* or a result of stress induced by the necessity to enforce wakefulness, particularly in the young pups. The regions with the highest P5 specific expression, the anterodorsal thalamic nucleus and lateral septum, are strongly responsive to early life stressors (Rivarola et al., 2008; Shin et al., 2018). Thus, differential effects of the stress associated with forced wakefulness may contribute to the developmental differences in these regions. However, the majority of regions, even in the same pathway, are not activated by the same stressors, suggesting that our manipulations largely capture the development of cFOS activation induced by wakefulness, though the necessity to enforce wakefulness at all ages must be kept in mind when interpreting our results.

### Early activation of the egocentric monitoring systems

We identified a number of areas with consistent cFOS expression at the earliest timepoint (P5). The postsubiculum L4-6, posterior hypothalamic nucleus, supramammillary nucleus, entorhinal cortex (L2), and dentate gyrus all had early expression patterns that were similar to, but lower in intensity than, their adult pattern. Additionally, the anterodorsal thalamic nucleus expressed cFOS at higher levels at P5 and P9 than the adult, while the septum showed expression at all ages. Together, these regions can be roughly clustered into the “head-direction circuit” (Taube, 2007) and the “ascending brainstem-hippocampal synchronizing pathway” that modulates theta-band oscillations (~4-10 Hz) (e.g., Kowalczyk et al., 2021) and their initial points of entry to the entorhinal and hippocampal cortex.

Head-direction information from vestibular and proprioceptive inputs is transmitted by the anterodorsal thalamic nucleus to postsubiculum L4-6, where it is integrated with cortical sensory inputs (including visual and auditory) and motor efferent copies. This information is passed by the postsubiculum L2-3 back to the anterodorsal thalamic nucleus and onward to the upper layers of the entorhinal cortex where it is incorporated into the firing of the grid neurons. Adult-like directional coding is present in the anterodorsal thalamic nucleus and postsubiculum L4-6 of rats by P12, before eye opening (Tan et al., 2015). Vestibular, rather than visual, cues thus appear to prime the development of the head-direction circuit (Valerio and Taube, 2016; Tan et al., 2017). The early cFOS expression we observe in these areas may reflect an important role for the head-direction system in navigating the blind pup toward the mother using olfactory information (Al Aïn et al., 2013; Tan et al., 2017). We identified several olfactory areas including the olfactory bulb, anterior olfactory nucleus, and piriform cortex as showing cFOS expression by P5 (Pompeiano and Colonnese, unpublished data). Inputs from the medial septum (Viney et al., 2018) might also contribute to the early activation of postsubiculum L4-6. The output layers of postsubiculum, L2-3, develop cFOS expression later, by P13, possibly beginning the transfer of processed head-direction information to entorhinal cortex L2-3 (Preston-Ferrer et al., 2016; Honda and Furuta, 2019) and contributing to the increase in cFOS expression in this structure at this age. Postsubiculum L2-3 receives inputs from multiple areas, including those mediating the theta rhythm (medial septal complex; Viney et al., 2018), medial entorhinal cortex L2-3 (van Groen and Wyss, 1990b), and dorsal CA1 that may, therefore, play a role in the onset of postsubiculum L2-3 activation.

Visual information anchors the head-direction circuitry to the surroundings and reaches all stations of the circuitry with different effects on their cFOS levels. Interestingly, the onset of vision appears inconsequential for cFOS expression at P13. Visual cortical areas that project to postsubiculum L2-3 as well as the retrosplenial cortex that inputs to postsubiculum L4-5 (Taube, 2007; Yoder et al., 2011) express cFOS only after P13 (Pompeiano and Colonnese, unpublished data). Further maturation of visual inputs may contribute to the later cFOS activation in the retrosplenial cortex and maturation in both laminas of the postsubiculum. The anterodorsal thalamic nucleus, that receives visual information through postsubiculum L5-6 (Yoder and Taube, 2011) and retrosplenial cortex L6a (Sripanidkulchai and Wyss, 1987) as well as direct visual inputs from the optic nerve (Sefton et al., 2015), actually loses cFOS expression as visual inputs come online (Colonnese et al., 2010).

The other major early expressing areas—the posterior hypothalamic nucleus, supramammillary nucleus, and septum—are part of the theta-modulating system (Kowalczyk et al., 2021) and are strongly implicated in the regulation of neural processing by internally and externally mediated positive and negative states (Tsamis et al., 2020; Wirtshafter and Wilson, 2021). The medial septum shows very low cFOS expression even in adult awake rats (Pompeiano et al., 1994; Cirelli et al., 1995) and mice (“sleep study”; Allen Institute for Brain Science, 2008) as a result, at least in part, of tonic inhibition by the supramammillary nucleus (Aranda, 2016), making conclusions about its activity difficult. Theta activity can be induced in the posterior hypothalamic nucleus of rats at P8, the earliest day examined, and this activity increases in incidence and amplitude up to P24 (Caban et al., 2018). The lateral septum (intermediate and ventral parts) is remarkable because it has among the strongest expression of any region examined here at P5. This occurs before any substantial activation is seen in the ventral hippocampus, the main input source. However, the staining partly occupies areas expressing encephalin and neurotensin and receiving inputs from the lateral hypothalamic area and posterior hypothalamic nucleus (areas r.dl.m and r.vl.v in Risold and Swanson, 1997), which do express cFOS at P5 (Pompeiano and Colonnese, preliminary observations). The lateral septum controls theta rhythms through projections to the medial septum (Wirtshafter and Wilson, 2021) and also makes strong hippocampal inputs and connections with subcortical areas which are topographically organized (Risold and Swanson, 1996, 1997; Deng et al., 2019; Rizzi-Wise and Wang, 2021; Wirtshafter and Wilson, 2021). Coherent theta during movement is not observed in the hippocampus until P10 in rats (Leblanc and Bland, 1979); thus, the early expression of cFOS in these regions likely does not indicate mature-like activity in these regions. However, it may suggest that early activation primitives of theta may be present in the theta-modulatory system and contribute to early hippocampal activation or that important integrative functions of the region, such as activation during arousal and locomotion (Risold and Swanson, 1997; Wirtshafter and Wilson, 2021), are present early and available to the hippocampus and regions of cortex.

### Entorhinal/hippocampal expression progression follows the tripartite circuit

cFOS is first seen in entorhinal L2 and dentate gyrus (by P5), followed by CA3 and entorhinal L6 (by P13), and, finally CA1, entorhinal L5 and L3 (by P17) (Table 2). By considering also the time when adult-like levels are reached (Table 2), it appears that activation follows the flow of information through the stations of the main entorhino-hippocampal circuit, rather than the time of birth of the neurons or the developmental gradients of cellular maturation.

**Table 2:** cFOS maturation follows the tripartite circuit activity flow.

**Age**	**Brain areas**							
	**Ent L2**	**DG**	**CA3**	**CA1**	**Ent L3**	**Ent L5**	**VS**	**DS**
Activation start	P5**→**	P5**→**	P13**→**	P17**→**	P17	P17	P5	Ad
Activation at adult level	P5**→**	P9**→**	P13**→**	Ad	**←**P17	P17	P9	Ad

Arrangement of the entorhinal cortex (Ent) and hippocampal areas in order of their position in the tripartite circuit (green arrows) is congruent with age of first significant cFOS staining (top row). The age at which they reach adult levels of expression is violated by CA1 which has very high levels in adults, some of which may be mediated by late development of integration from Ent L3 (backward arrow; bottom row). Maturation of ventral subiculum (VS) is early, following that of the dentate gyrus (DG), while dorsal subiculum (DS) is late and does not have significant expression until adulthood.

Early activation of the entorhinal cortex L2 is likely due to the arrival of inputs from the olfactory bulb as olfactory input to the lateral entorhinal cortex provides a significant component of the coordinated activity during the second post-natal week (Gretenkord et al., 2019) and we observed strong cFOS expression in this region (Pompeiano and Colonnese, unpublished data). The other major sources of identified drive to the neonatal entorhinal cortex and hippocampus, spontaneous muscle twitches which trigger early sharp waves (Cossart and Khazipov, 2022), are an unlikely source of activation as both twitching and sensory inputs are suppressed by waking during the first 2 post-natal weeks (Del Rio-Bermudez and Blumberg, 2022). Somatosensory activity may enter the entorhinal cortex through the head-direction and theta-rhythm circuits as these regions express relatively high levels of cFOS at P5 and target entorhinal upper layers (Moser et al., 2018; Somogyi and Klausberger, 2018). The early activation of entorhinal L2 likely involves stellate/fan neurons which mature early (Wills et al., 2010). L2 does not have head-direction cells, but its stellate cells later develop into grid cells which display covert head-direction information (Moser et al., 2018; Gerlei et al., 2020).

The dentate gyrus shows cFOS at P5, possibly driven by convergence of inputs from entorhinal L2 (its stellate/fan neurons massively project to the dentate gyrus; Moser et al., 2018) and input from the theta-related regions, the supramammillary nucleus, and medial septum (Cappaert et al., 2015; Hashimotodani et al., 2018; Chen et al., 2020). These single inputs increase the firing of dentate granule cells when paired (Mizumori et al., 1989; Hashimotodani et al., 2018). Staining in the dentate gyrus starts in the dorsal blade and extends to the ventral blade only later (by P13) reflecting the maturation timing of the two blades (Tamamaki, 1999). cFOS-positive cells are first seen in the granule cell layer, at the border with the molecular layer, a location where the neurons mature first as indicated by the loss of immature markers (e.g., DCX and NeuroD1) and gain of mature markers (e.g., Prox1, and NMDA receptor subunits N1 and N2A) (Allen Institute for Brain Science, 2008; Gustorff et al., 2021).

CA3 expression by P13 follows the increased expression of the dentate gyrus, while the activity of the entorhinal L2 and medial septum may also contribute (for the anatomy, see Cappaert et al., 2015; Moser et al., 2018). Expression in CA1 seems to closely follow CA3, suggesting that the CA3-CA1 network may already form a coherent system in pups as it does in adults (Buzsáki and Fernández-Ruiz, 2018). Among other afferent areas to CA1 (Cappaert et al., 2015), postsubiculum L2-3 and the most anterior part of the lateral entorhinal L3 may contribute to CA1 initial activation as they also express cFOS on P13-P17. Entorhinal L3 electrophysiological responses to postsubiculum are immature at P9/P10 and adult-like at P14/P15, and they increase until P28-P30 (Canto et al., 2019), in perfect agreement with our cFOS data from L3.

The pattern of cFOS expression in CA1 is nearly adult-like by P13 but becomes significantly denser through adulthood. Dense cFOS expression is thus correlated with the dissolution of early activities such as the early sharp waves and GDPs and the onset of ‘experience-dependent' local and distal activity (Cossart and Khazipov, 2022). For example, hippocampal network oscillations are modulated by theta rhythms starting from P8 but only become full 8 Hz oscillations in the third post-natal week. In rats, adult sharp wave ripples do not appear until after P12 (Cossart and Khazipov, 2022). Thus, full cFOS staining appears to reflect the onset of these mature activity patterns and the connection of the hippocampal formation to the outside world. The pattern of this onset reflects the external flow of information from entorhinal L2-3:DG:CA3:CA1:entorhinal L5-6, rather than the endogenous flow of spontaneous activity from CA3:CA1 & DG, or the sharp-wave/ripple network originating in CA2 and excluding the DG.

L5a of the entorhinal cortex is the last layer to express cFOS (after P17). L5a receives local projections from L5b (Ohara et al., 2021). Its large pyramidal neurons also extend their apical dendrites to the superficial layers therefore reaching olfactory, perirhinal, postrhinal, and postsubiculum inputs (Moser et al., 2018) which appear unable to trigger cFOS in these neurons at earlier times. L5a late activation may be due to the integrative capacities of its neurons (Canto et al., 2008) and an increased activation seen in multiple afferent areas, including perirhinal and postrhinal cortices and postsubiculum.

Maturation of the different layers of the entorhinal cortex occurs over a prolonged period of time and suggests a specific order in which mature information may originate from the entorhinal cortex (for the anatomy, see Cappaert et al., 2015; Moser et al., 2018). The perforant path initially bringing head-direction information (later incorporated into grid cells) from *L2* to the dentate gyrus/CA3 is activated first (by P5). It is followed by activation of *L6* projections to anterior thalamic nuclei that possibly contain head-direction information integrated with hippocampal and prefrontal information (by P9) and later also with visual information. By eye opening, information from the head-direction cell together with visual information travels from *L3* to CA1/dorsal subiculum, and finally (after P17), a highly integrated output from *L5a* reaches cortical and subcortical areas. Our data do not allow us to speculate about other types of space-related information generated in the entorhinal–hippocampal circuitry.

### Ventral and dorsal subicula are activated at very different times

The dorsal hippocampus is traditionally associated with spatial memory and navigation while the ventral hippocampus with emotion-driven memory and behavior (Fanselow and Dong, 2010; Strange et al., 2014). The subiculum plays a role in encoding and retrieving these memories (Ding, 2013). Dorsal and ventral subicula receive their main input from the dorsal and ventral CA1, respectively, and provide a primary output from the hippocampus to cortical and subcortical areas (Cappaert et al., 2015). Interestingly, both dorsal and ventral hippocampus express cFOS by P9 together with the ventral subiculum. These observations agree with the finding of place fields across the dorsoventral axis of the hippocampus with those in the ventral hippocampus being modulated by anxiety during spatial navigation (Malagon-Vina et al., 2023) and with the finding that cFos developmental transcription in rat hippocampus is significantly correlated with developmental age and not with the hippocampal portion (Signature 2 in Figure 1B and Supplementary material 1 in Olsen et al., 2023). The dorsal subiculum does not have significant cFOS expression until after P17, possibly due to the fact that it is strongly integrative (Wyss and Van Groen, 1992; Ding, 2013), as part of its role in distributing navigation-associated information (speed, trajectory, and place information) to areas including the retrosplenial cortex, accumbens nucleus, and medial mammillary, anteroventral thalamic, and reuniens nuclei (Kitanishi et al., 2021). The late expression of cFOS in the dorsal subiculum may thus reflect the acquisition and refinement of such information through prolonged experience and reflect a requirement for strong CA1 activation available only in adulthood. Interestingly, the thalamic and hypothalamic targets of the dorsal subiculum such as reuniens and anteroventral thalamic nuclei also show late cFOS expression.

### Activation in retrosplenial, perirhinal, and postrhinal cortices follows the onset of mature, continuous activation throughout the cortex

We additionally examined retrosplenial, perirhinal, and postrhinal cortices here because they make major reciprocal connections with cortical and hippocampal areas and are major points of information exchange between the two areas (van Groen and Wyss, 1990a, 2003; Furtak et al., 2007; Agster and Burwell, 2009; Sugar et al., 2011; Willems et al., 2016; Yamawaki et al., 2016; Aggleton et al., 2021). These regions express cFOS late, with robust expression across a majority of layers occurring near eye opening (P13). Hippocampal lesions cause a strong decrement of cFOS staining in all retrosplenial layers (Albasser et al., 2007; Mao et al., 2018), and thus, it is likely their development waits for robust input from CA1 and entorhinal cortex L5 (Wyss and Van Groen, 1992; Sugar et al., 2011; Gao et al., 2021). In general, the retrosplenial cortex integrates spatial cognition including navigation and memory (Aggleton et al., 2021; Stacho and Manahan-Vaughan, 2022). Area 29 deals with both visual and non-visual cues, including proprioceptive cues, while area 30 deals mainly with visual cues (Aggleton et al., 2021). Area 30 is delayed in its pattern of cFOS expression compared to area 29, perhaps as a result of the delayed input from visual regions compared to non-visual. The earliest activation in both regions was seen in L6b which likely includes subplate neurons (Kanold and Luhmann, 2010). In the sensory cortex, subplate neurons are the first neurons to be generated, to respond to sensory stimuli (Wess et al., 2017), and to express cFOS (Pompeiano and Colonnese, unpublished data), but their role has not been explored in the retrosplenial cortex. It is tempting to speculate that early inputs from the anterodorsal thalamic nucleus may drive early activation in area 29 L6b. In fact, L3 contains head-direction cells (Aggleton et al., 2021) and receives abundant inputs from the anterodorsal thalamic nucleus and postsubiculum dorsal layers (van Groen and Wyss, 2003; Sugar et al., 2011; Aggleton et al., 2021), which may make collaterals to subplate neurons early as in sensory regions.

Other integrative cortical regions with strong hippocampal connections, the perirhinal and postrhinal cortices, are also late developing. They play a role in object recognition and contextual information processing (Sethumadhavan et al., 2020; LaChance and Taube, 2022) and show cFOS expression following object recognition tasks (Cinalli et al., 2020; see also Sethumadhavan et al., 2022) and novel context exploration (Kinnavane et al., 2017), respectively. The perirhinal cortex shows activation in most layers by P13 with the remaining L5a showing cFOS by P17, when expression became stronger in area 36 than 35. Area 36 receives more polymodal and less olfactory afferents than area 35 (Furtak et al., 2007). The postrhinal cortex shows a similar progression of activation across its layers. Similarly to entorhinal, retrosplenial, and perirhinal cortices, postrhinal L5a is the last one to show cFOS expression (after P17) possibly reflecting integrative properties of neurons in this layer. Overall, these cortical regions seem to follow the pattern of primary cortical expression, where activation begins in the earliest generated and connected regions, L6 and subplate, and only later is seen in L3 and 5 before adding the major thalamic input layer (L4) last when mature patterns of continuous activity are widespread in hippocampus and cortex (Colonnese and Phillips, 2018).

### cFOS vs. other markers

Recently, Donato et al. (2017) suggested an order of maturation in the entorhinal–hippocampal circuit based on the disappearance of doublecortin, a widely used marker of post-mitotic immature neurons (Friocourt et al., 2003). The same order and timing of maturation were suggested by parvalbumin expression, a marker of interneuron maturation (Donato et al., 2017). These markers appear to signal a different maturation gradient than ours. Doublecortin disappears much later than cFOS appears (even considering the timing of rat and mouse). The order of maturation is also different. For example, DG matures after CA1 in Donato et al. (2017) but expresses cFOS before CA1. Doublecortin is co-expressed with markers of neuronal maturation such as NeuN (Duan et al., 2016) both during development (Boekhoorn et al., 2014; Cahill et al., 2017) and in newly generated neurons (Brown et al., 2003), as well as with phosphorylated CREB (Balta et al., 2018) and cFOS (e.g., Barr and Unterwald, 2015), and thus likely reflect the extended maturation of the dentate gyrus neurons (Abbott and Nigussie, 2019). These observations suggest that functional maturation and structural maturation are not always correlated, and combined use of these markers could reveal additional information.

cFOS expression may start when neurons acquire a mature glutamate receptor-mediated postsynaptic neuronal response (Vendrell et al., 1998; Lai et al., 2016). In the hippocampus, the expression of the four AMPA receptor subunits, GluN1 and GluN2B NMDA receptor subunits, as well as CaMKIIα is very low at P2, it increases by P7, and it strongly increases thereafter reaching adult-like levels by P14 (in rats; Osten et al., 2007). The GluN2A NMDA receptor subunit is expressed at later times, with levels becoming significant by P14 and increasing to mature levels by P28 (in rats; Osten et al., 2007). The temporal expression pattern of cFOS in the hippocampus seems in good agreement with that of these glutamate-related markers.

## Conclusion

Here, we showed that cFOS immunohistochemistry is a promising candidate as a simple biomarker of activity maturation in the hippocampal–entorhinal network. Expression progressively increased both in the number of brain areas affected and in intensity during post-natal development in awake mouse pups in a stereotypical and reproducible pattern that appeared largely linked to the onset of mature background activity patterns including theta and gamma rhythms. In the hippocampus and entorhinal cortex, our data are compatible with a progressive activation along the trisynaptic circuit and the unidirectional flow of excitatory connections, rather than being determined by birth date or cellular maturation. cFOS staining was useful to identify potential contributors to early activity such as the anterodorsal thalamic nucleus and lateral septal nucleus that express state-dependent ‘activation' at the earliest ages.

## Data availability statement

The raw data supporting the conclusions of this article will be made available by the authors, without undue reservation.

## Ethics statement

The animal study was reviewed and approved by George Washington University IACUC.

## Author contributions

MP contributed to data collection and prepared figures. MP and MC contributed to the conception and design of the study, wrote the first draft of the manuscript, contributed to manuscript revision, read, and approved the submitted version. All authors contributed to the article and approved the submitted version.
